# Synergistic Effect of Cobalt/Ferrocene as a Catalyst for the Oxygen Evolution Reaction

**DOI:** 10.1021/acs.jpclett.4c02039

**Published:** 2024-10-15

**Authors:** Jose M. Abad, Alba Duprat-Alvaro, Raquel Sainz, María Victoria Martínez-Huerta, Marcos Pita, Antonio L. De Lacey

**Affiliations:** Instituto de Catálisis y Petroleoquímica, CSIC, C/Marie Curie 2, 28049 Madrid, Spain

## Abstract

There is a great deal of interest in the development of electrocatalysts for the oxygen evolution reaction (OER) that are stable and have high activity because this anodic half-reaction is the main bottleneck in water splitting and other key technologies. Cobalt and iron oxide and oxyhydroxide electrocatalysts constitute a cheaper alternative to the highly active and commonly used Ir- and Ru-based catalysts. Most of the described electrocatalysts require tedious synthetic and expensive preparation procedures. We report here a facile and straightforward preparation of an electrocatalyst by a combination of commercial compounds, such as cobalt chloride and ferrocene. A highly active and stable OER electrocatalyst is obtained, which shows a low overpotential in the alkaline medium as a consequence of a synergistic effect between both compounds and is inexpensive.

## Graphical abstract

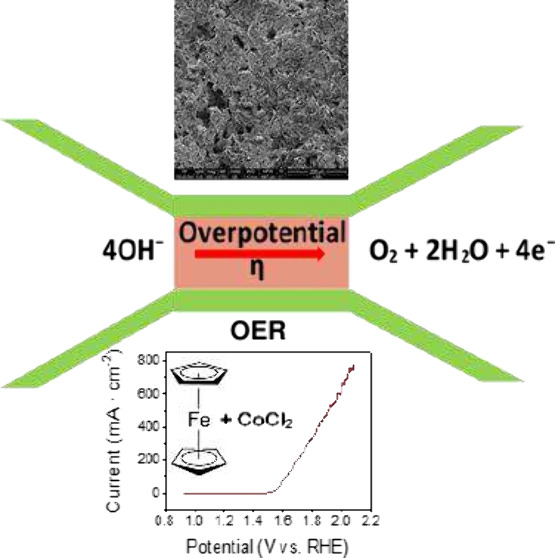

There is a great deal of interest in decreasing the consumption of energy that originates from unsustainable fossil fuels and the CO_2_ emissions produced.^1^ In this sense, clean and renewable energy sources, such as solar and wind, are attractive sustainable and environmentally friendly alternatives. However, one challenge is to transform the energy derived from these intermittent sources into storable and transportable fuels. Electrochemical hydrogen production has been widely investigated due to its ability to both store chemical energy and further release it for electricity production in fuel cells.^2–6^

Typically, H_2_ is produced via the hydrogen evolution reaction (HER) at the cathode by electrolytic water splitting in addition to the simultaneous anodic oxygen evolution reaction (OER).^7,8^ However, the OER is often regarded as the main bottleneck in water splitting due to its slow kinetics, which limits the efficiency of the energy conversion. This anodic half-reaction, which is crucial in water electrolysis, metal–air batteries, and electrochemical CO_2_ reduction, occurs via a four-electron–proton-coupled pathway (2H_2_O → O_2_ + 4H^+^ + 4e^–^, acidic conditions) (4OH^–^ → O_2_ + 2H_2_O + 4e^–^, alkaline conditions) that requires application of a high overpotential to overcome the kinetic barrier.^9,10^ To overcome it, research in this field has been focused on the development of low-cost, highly active, and stable electrocatalysts to make the OER feasible by dismissing the overpotential.^11–15^

Most commonly used catalysts involve noble metals such as iridium­(IV) or ruthenium­(IV) oxides, which to date are still the benchmarks for OER catalysts.^16–18^ However, they are scarce and expensive metals for large-scale applications, and Ru- or Ir-based materials normally suffer from serious stability issues, especially under harsh water electrolysis conditions.^18,19^ As an alternative, transition metals (Fe, Co, Ni, and Mn) and their oxides have been extensively explored as they can participate in high-performance electrocatalysis and are cheaper to obtain.^10–25^

Because the electrochemical abilities of oxides are related to their structural properties, numerous advances have been made in designing and producing new combinations of these transition metal oxides, although these processes sometimes involve tedious synthetic procedures.^26,27^ Here, we report, as a novelty, a very easy and straightforward method of preparation and performance of an electrocatalyst for the OER in alkaline media formed by the combination of Co and Fe into one material deposited on a carbon electrode, leading to a synergistic effect from a minimal loading of ferrocene (Fc) and cobalt­(II) chloride. Both cobalt and iron oxide and oxyhydroxide electrocatalysts have been regarded as strong candidates for OER catalysis, with high activity from neutral to alkaline media.^25–33^ Furthermore, Fc has also been used in some recent works combined with cobalt, such as ferrocene-based bimetallic CoFe–FcDA nanosheets,^34^ ferrocene-incorporated cobalt sulfide nanoarchitectures,^35^ or cobalt- and ferrocene-based metal–organic frameworks,^36^ showing an efficient OER.

In this work, catalysts were prepared by simple drop-casting on porous graphite electrodes (3 mm diameter) of a tiny amount of a catalytic ink (4 μL) containing CoCl_2_·6H_2_O (36 μg, 0.5 mg cm^–2^) and ferrocene (18 μg, 0.25 mg cm^–2^) as the only metals mixed with activated carbon Vulcan XC-72R and Nafion. These minimal loadings were found to be optimal after optimization studies carried out with different loadings of ferrocene and cobalt to estimate which gave the best performance in terms of obtaining a low OER overpotential and high catalytic activity (Figures S1 and S2).

Co^2+^ from the CoCl_2_ is expected to react in an alkaline medium with hydroxyl ions forming cobalt hydroxide [Co^2+^ + 2OH^–^ ⇆ Co­(OH)_2_].

This compound can be transformed into stable oxide Co_3_O_4_ by oxidation [3Co­(OH)_2_ + 2OH^–^ ⇆ Co_3_O_4_ + 4H_2_O + 2e^–^]. Electrochemical characterization of the prepared electrocatalysts was performed under stationary conditions in a highly alkaline medium with a pH of 13.89, utilizing a 1 M KOH electrolyte at a potential window prior to the OER. Figure 1a depicts cyclic voltammograms (CVs) recorded in 1 M KOH from 0.93 to 1.42 V versus RHE at a scan rate of 100 mV s^–1^ for a Co/Fc-modified anode. The first ongoing scan shows two voltammetric anodic peaks at 1.25 and 1.15 V, which are tentatively associated with Co-related reactions, where Co­(II) is gradually oxidized to Co­(III), because they are not observed in the CV for the electrode containing only Fc (Figure 1c). These peaks are in agreement with those reported in the literature for oxidation and transformation of Co_3_O_4_ and Co­(OH)_2_ hydroxide to hydrous oxide CoOOH [Co_3_O_4_ + H_2_O + OH^–^ ⇆ 3CoOOH + e^–^ and Co­(OH)_2_ + OH^–^ ⇆ CoOOH + H_2_O + e^–^, respectively].^37–43^ In successive scans, the peak at 1.25 V disappears, which indicates that the initial species, Co­(OH)_2_, is not being regenerated but transformed into Co_3_O_4_. Simultaneously, the peak at 1.15 V decreases, which has been reported to be due to the poor diffusion of the electrolyte through the film on the time scale of the experiment, preventing the reduction of all of the cobalt­(III) sites in the film.^44^ This results in the establishment of a well-resolved Co­(II)/Co­(III) redox wave corresponding to the conversion between Co_3_O_4_ and CoOOH as described above, CoOOH being the active site for further water oxidation (CoOOH + OH^–^ ⇆ CoO_2_ + H_2_O + e^–^).^37,42–44^ This pair of quasi-reversible peaks has a half-wave potential, *E*_1/2_ (Ep_c_ + Ep_a_)/2, at a scan rate of 100 mV s^–1^ of 1.06 V, where Ep_c_ and Ep_a_ are the cathodic and anodic peak potentials, respectively, with a peak-to-peak separation (Δ*E*_p_ = Ep_a_ – Ep_c_) of ∼59 mV, and the peak currents are proportional to the scan rate, a typical property of redox adsorbed species (Figure S3). Integration of the total charge of the Co­(II)/Co­(III) redox anodic wave gave a surface coverage of Co_3_O_4_ of 2.4 × 10^–11^ mol cm^–2^.

**Figure 1:**
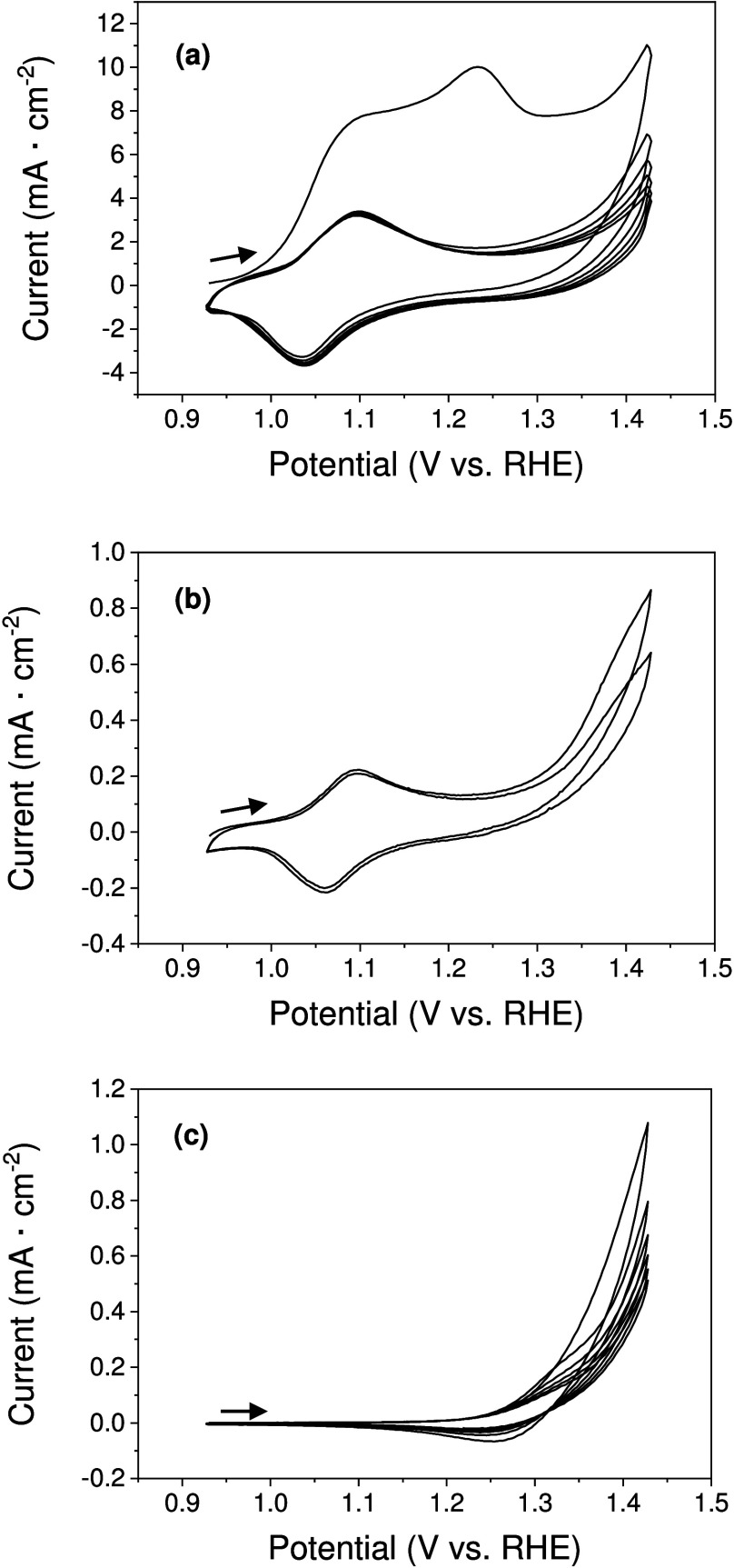
Cyclic voltammograms in a 1 M KOH solution of a Co/Fc-modified electrode at scan rates of (a) 100 and (b) 5 mV s^–1^ and (c) the Fc-modified electrode as the control, at a scan rate of 100 mV s^–1^.

The CV at a slow scan rate (5 mV s^–1^) for the Fc/Co-modified electrode in Figure 1b also shows a preoxidation wave at 1.4 V associated with Fc because it is the only redox wave measured for the Fc-modified electrode (Figure 1c). This wave disappeared in successive scans. It might be attributed to a formed mixed metal Co/Fe hydroxide in the resting state and its oxidation to amorphous Co/Fe oxyhydroxide [Co­(Fe)­OOH] at high redox potentials.^28,45^

X-ray photoelectron spectroscopy (XPS) was used to obtain further information about the surface of the electrocatalysts. Three Fc/Co catalyst electrodes were analyzed: EL1, as-prepared catalyst-modified electrode before any electrochemical process; EL2, after CVs were recorded as depicted in Figure 1a; EL3, after further OER performance evaluation. The measurements were challenging due to the low content of Co and Fe; thus, a large number of measurements were taken to maximize the signal-to-noise ratio. The spectra obtained were deconvoluted to gain information about redox intermediate species generated during the electrochemical processes. High-resolution Co 2p spectra (Figure 2a) show spin–orbit splitting into 2p_1/2_ and 2p_3/2_ components. For EL1, the Co 2p_3/2_ spectrum shows one main peak located at 781.0 eV and a wide satellite peak at 787.5 eV. This spectrum could be assigned to Co­(II) from Co­(OH)_2_. For EL2 and EL3, in addition to the peak at 781 eV and the broad satellite peak at 787 eV, a new peak is observed at ∼780 eV of BE. This latter peak can be assigned to Co­(III) species (i.e., CoOOH or hydrated Co_2_O_3_), being more intense for the EL3 sample as consequence of a major conversion of Co­(II) to Co­(III) after OER performance (Table S1).^46–48^

**Figure 2:**
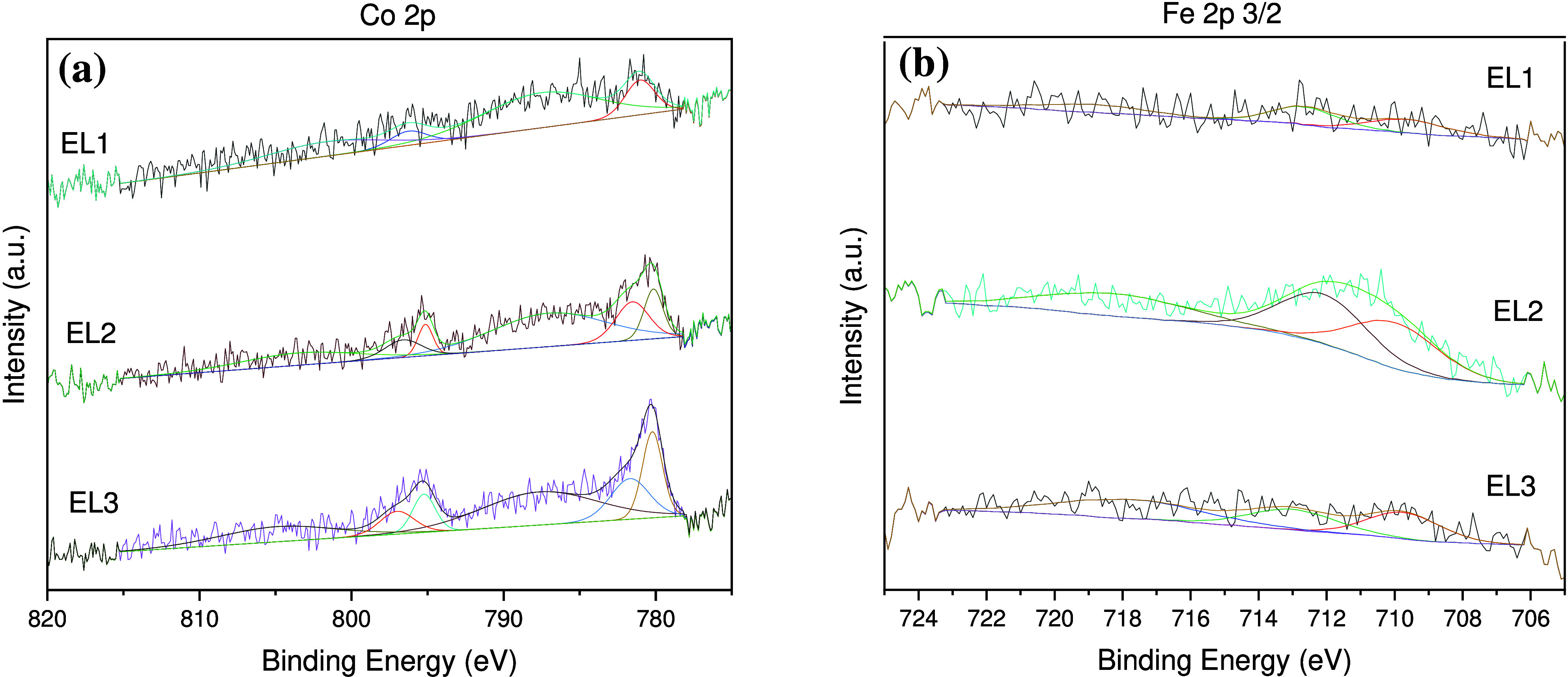
High-resolution XPS spectra for Fc/Co-modified electrodes of the (a) Co 2p energy region and (b) Fe 2p energy region. Abbreviations: EL1, as prepared before any electrochemical process; EL2, after cyclic voltammetry as depicted in Figure 1a; EL3, after further OER.

The XPS spectra of the Fe 2p energy region show two peaks around 710 and 712 eV, in addition to a satellite peak at 718 eV for all three electrodes (Figure 2b). They are typical peaks of Fe 2p_3/2_ in which the energy separation between the Fe 2p_3/2_ peak (710.8 eV) and the satellite peak was 8 eV (Table S2). This value is consistent with the Fe­(III) oxidation state as previously reported for films of iron oxyhydroxide,^28,33^ which is higher than the expected value of 5 eV for Fe­(II) ions.

The surface morphologies of the as-prepared Co/Fc catalyst-modified electrode and those after the OER were characterized by scanning electron microscopy (SEM). The SEM images in both cases show a porous structure on the surface in which in some areas can be distinguished Vulcan XC-72 carbon particles impregned by the Co/Fc catalyst (Figure S4). No significant morphological changes are observed after the OER process. The molar ratio of cobalt to iron obtained by energy-dispersive X-ray spectroscopy (EDX) analysis in different areas was 4.8 after the OER (Figure S5). Electrochemical evaluation of the OER activities for the Co/Fe-modified electrodes was carried out by linear sweep voltammetry measurements. The following reaction of oxyhydroxide is postulated to proceed:^10,23,29,49^

$$M^{3+}\left(O\right)\mathrm{-O}H+{OH}^{-}\left(aq\right)↔M^{2+}\mathrm{-O}H+O_{2}\left(g\right)+e^{‐}$$

Figure 3 shows a comparison of the results of linear sweep voltammetry (LSV) from 0.93 to 2.08 V versus RHE obtained for different configurations of the modified electrodes: (a) with a Co/Fc mixture, (b) without Fc, (c) without Co, or (d) with only Vulcan. As one can see, the Co/Fc catalyst provided the highest current density characterized by an onset η (0.24 V) and overpotential η@10 mA cm^–2^ (0.31 V) that were lower than those obtained for Co (η_onset_ = 0.28 V; η@10 mA cm^–2^ = 0.4 V), Fc (η_onset_ = 0.37 V; η@10 mA cm^–2^ = 0.46 V), or Vulcan XC 72R (η_onset_ = 0.4 V; η@6 mA cm^–2^ = 0.69 V) as supported electrocatalysts. These results clearly indicate that there is a synergistic effect in the bimetallic catalyst, which can be attributed, as mentioned above, to the formation of Co/Fe oxyhydroxide [Co­(Fe)­OOH].^45,50^ For comparison, an IrO_2_ oxide electrocatalyst was also prepared under the same conditions in terms of the amount deposited on the electrode. It displayed an overpotential higher than that obtained for the Co/Fc catalyst (Figure S6).

**Figure 3:**
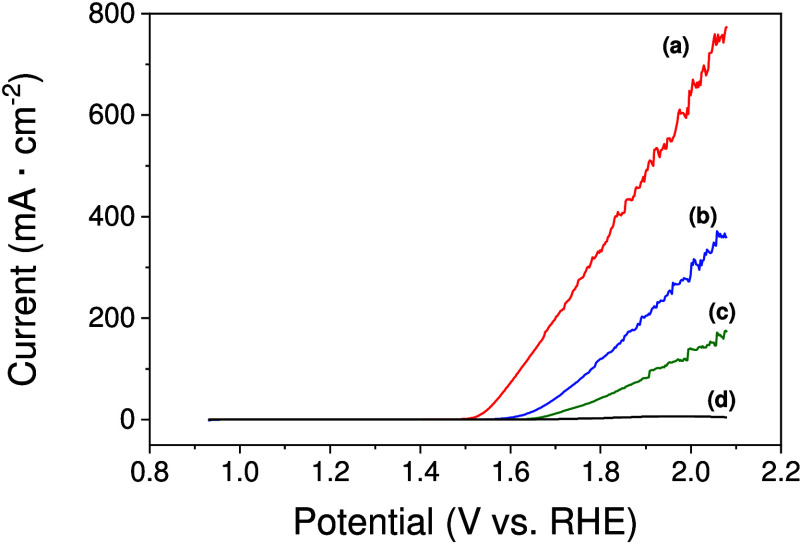
Linear sweep voltammograms in a 1 M KOH solution of the (a) Co/Fc-modified electrode, (b) Co-modified electrode, (c) Fc-modified electrode, and (d) only Vulcan-modified electrode. All graphite electrodes were prepared by drop-casting of an ink containing the different catalyst configurations mixed with activated carbon Vulcan and Nafion as described in the Supporting Information. The scan rate was 5 mV·s^–1^.

Electrochemical impedance spectroscopy (EIS) was carried out to estimate the electron transfer resistance (*R*_et_). Figure S7 depicts the Nyquist plots for the different compositions of the electrocatalyzers. A clear trend can be observed upon the addition of the different catalysts. The presence of Co species decreases the *R*_et_ to ∼30 Ω in the absence or presence of ferrocene (panel a or b, respectively, of Figure S7), indicating that the improvement is mainly due to the Co and not to Fc. Indeed, Fc in the absence of Co species also improves the *R*_et_ but to a lesser extent, *R*_et_ reaching ∼440 Ω, 14-fold higher than in the presence of Co. However, these values are also ∼4 times better than that of the Vulcan carbon electrode (∼1600 Ω). The improvement is attributed to a better interaction with the OH^–^ anionic species upon addition of the catalysts, which is greatly improved when Co^2+/3+^ is present. EIS measurements also provided data regarding the uncompensated resistance values (*R*_u_).^49^ The Co/Fc catalyst showed an *R*_u_ (8.5 Ω) close to that of Co (9.9 Ω) and lower than those of Fc or Vulcan catalysts, which had high resistances. Taking the determined *R*_u_ value, we compensated the *iR* drop for the previously acquired LSV data of the Co/Fc-modified electrode (Figure S8). Finally, Tafel analysis of the OER was performed by chronoamperometry measurements to gain information about the inherent kinetics of the Co/Fc electrocatalyst (Figure 4). Tafel slopes obtained by this method reflect the intrinsic catalytic activity of the material without any contribution from the capacitance current and at a true steady state, unlike the slopes derived from conventional voltammetry methods.^50^ A Tafel slope value of 54 mV decade^–1^ was obtained, which is smaller than that expected for Tafel slopes of Fe and Co oxides and hydroxides, ranging mostly between 60 and 120 mV decade^–1^ (in alkaline medium), and similar to that reported for Co_3_O_4_.^51^ This indicates that the Co/Fc electrocatalyst prepared in this work can promote faster kinetics for the OER process, achieving the required current density at lower or similar overpotentials in comparison to other efficient bimetallic CoFe catalysts recently reported in the literature.^11,25^ On the contrary, the ferrocene group appears to have a significant impact on the activity. Density functional theory calculations conducted by Xie et al.^34^ concluded that ferrocene units can act as mediators to facilitate electron transfer, thus contributing to the outstanding intrinsic activity observed.

**Figure 4:**
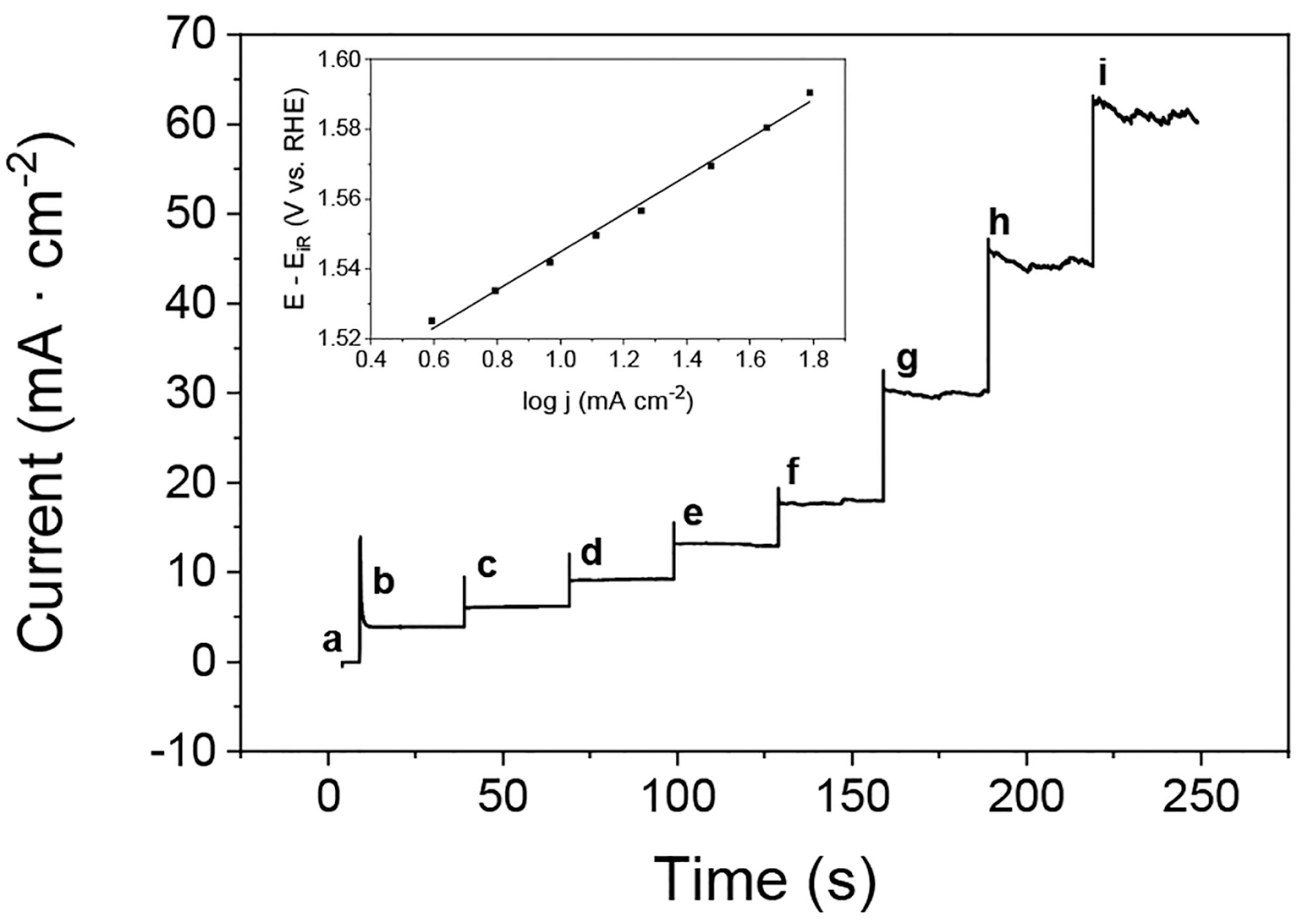
Chronoamperometry measurements of a Co/Fc catalyst electrode in 1 M KOH at potentials of (a) 0, (b) 1.528, (c) 1.538, (d) 1.548, (e) 1.558, (f) 1.568, (g) 1.588, (h) 1.608, and (i) 1.628 V. The inset shows a Tafel plot derived from current densities obtained as a function of the compensated potential applied.

The stability of the Co/Fc electrocatalyst was also evaluated. Figure 5 shows the results of a 24 h chronopotentiometry experiment at a current density of 10 mA cm^–2^. As one can see, the electrocatalyst provided a constant potential of ∼1.54 V, keeping the film stable during the assay period and not being affected by the generation and evolution of oxygen bubbles.

**Figure 5:**
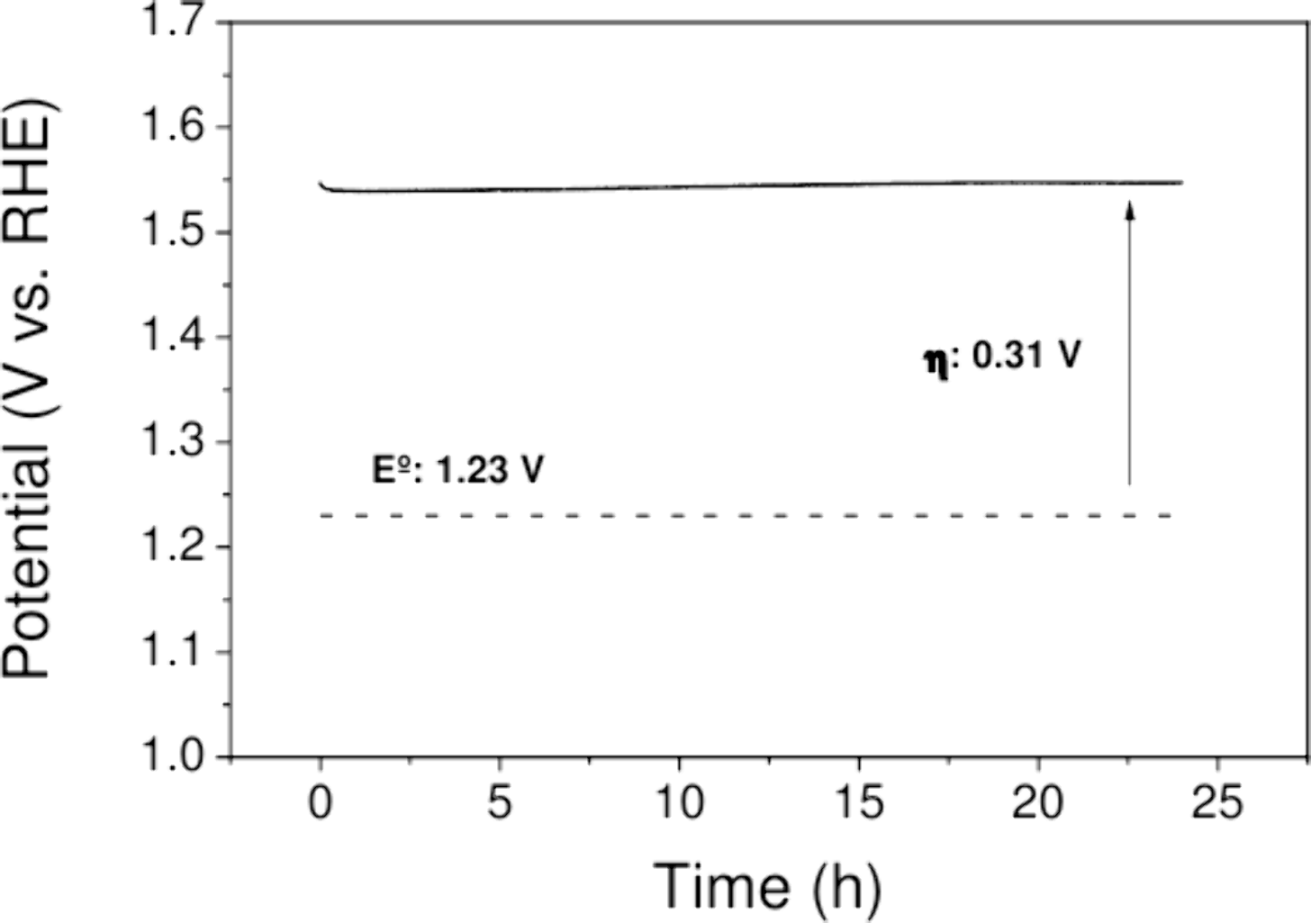
Chronopotentiometry stability test of the Co/Fc catalyst electrode for 24 h at 10 mA cm^–1^ in a 1 M KOH solution.

To summarize, we report the straightforward preparation of a Co/Fc electrocatalyst for the OER under alkaline conditions. The catalyst showed superior performance in terms of (i) a low overpotential of 310 mV at 10 mA cm^2^ and a Tafel slope of 54 mV decade^–1^, (ii) a high current density, and (iii) stability for >24 h. According to the figure of merit reported by Wang et al.^11^ for comparative analysis of OER catalysts based on their overpotentials and operational stabilities, our catalyst lies in the category of excellent. A synergistic effect between Co/Fc is observed because introducing ferrocene facilitates charge transfer during water-splitting reactions as a consequence of the inclusion of iron metal in addition to the cobalt, which provides stability. The method represents a simple and easy strategy for obtaining anodes that can be scaled up to real devices without the need for previous and tedious synthetic procedures. It is noteworthy that only commercial chemicals are used with a negligible cost because the amounts of metals, both cobalt and iron, employed are minimal (125 and 76 μg cm^–2^, respectively).

## Supplementary Material



## Notes

Experimental section, Figures S1–S8, and Tables S1 and S2 (PDF)
