# Supplementary material for: Synergistic Effect of Cobalt/Ferrocene as a Catalyst for the Oxygen Evolution Reaction
Source: J Phys Chem Lett. 2024 Oct 15;15(42):10638–43. doi: 10.1021/acs.jpclett.4c02039 (PMC12303365; doi:10.1021/acs.jpclett.4c02039)
Supplement: Supplementary file 1 [file jz4c02039_si_001.pdf]

Synergistic effect of cobalt/ferrocene as catalyst for oxygen evolution reaction

Jose M. Abad, Alba Duprat-Alvaro, Raquel Sainz, María Victoria Martínez-Huerta, Marcos Pita, Antonio L. De Lacey.*

Instituto de Catálisis y Petroleoquímica, CSIC. C/Marie Curie 2, 28049 Madrid, Spain.

Experimental section

Materials and reagents.

Graphite rods of 3 mm diameter, low density, 99.995% trace metals basis, cobalt chloride hexahydrate (ACS reagent), Ferrocene (98%) and Nafion™ perfluorinated resin solution (5 wt. %) were supplied by Sigma-Aldrich. Carbon black Vulcan XC-72R was supplied by Fuelcell company.

Electrode preparation and catalytic ink deposition

Graphite electrodes (0.071 cm^2 geometric area) were polished softly on a fine grit polishing pad (BASi), rinsed, and sonicated for 15 min in Milli-Q water. Catalytic ink was prepared by mixing 1.5 mg of carbon black Vulcan with 1 mL of ethanol and 0.25 mL of Milli-Q water and 5 µL of Nafion. The mixture was sonicated for 30 min in an ultrasound bath to form a homogeneous ink. Following, 20 µL of a 10 g · l^{-1} ferrocene ethanolic solution and 4 µL of a 100 g · L^{-1} cobalt chloride hexahydrate aqueous solution, previously prepared, were added to 20µL of the mixture. Ink deposition was carried out by drop-casting of 2 µL of this mixture onto the graphite surface and left drying for 5 min at room temperature. This deposition process was done twice and immediately the performance of the electrode was analyzed. The final amounts employed were $CoCl_2$ $6H_2O$: 36 µg, 0.5 mg·cm^{-2} and ferrocene 18 µg, 0.25 mg·cm^{-2}.

Electrochemical measurements

Electrochemical measurements were done with an Autolab potentiostat (PGSTAT 30, Eco Chemie) using a three-electrode cell in 1 M KOH solution, pH: 13.89, at room temperature. A graphite rod was used as

counter electrode, and the potentials were measured with respect to a mercury oxide (Hg/HgO) reference electrode (handling and disposal of the reference electrodes were carried out as recommended by local EPA rules). To facilitate analysis and comparison, all potential values obtained versus Hg/HgO were converted into potentials versus the reversible hydrogen electrode (RHE) using the Nernst equation as follows:

$$E_{RHE} = E_{Hg/HgO} + 0.0592pH + E^{o}_{Hg/HgO} = E_{Hg/HgO} + 0.928V$$

Where $E^{o}_{Hg/HgO}$ signifies the standard thermodynamic potential (0.1053 V) of Hg/HgO, $E_{Hg/HgO}$ is the potential vs. Hg/HgO obtained during electrochemical measurements, and E_{RHE} represents the potential vs. RHE.

The reported overpotential η is referred to a current density reaching 10 mA cm^{-2}:

$$\eta = E_{10\ mA\ cm^{-2}} - E^{o}\ (1.23V)$$

The chronopotentiometric stability test during 24 hours was performed at an applied current density of 10 mA· cm^{-2} in a 1 M KOH solution. The electrochemical impedance spectroscopy (EIS) was carried out for the prepared electrodes within the frequency range from 0.1 MHz to 1 Hz, employing an AC potential amplitude of 10 mV and a bias potential of +1.65 V vs RHE. The resulting Nyquist plots were further analyzed to assess the resistance of the electrocatalytic materials.

Tafel analysis of OER was carried out by chronoamperometry measurements restricting the steady state experiment within the first few decades of low current density. The interval between two step potentials was 0.01 – 0.02 V and the potential was corrected for iR drop before plotting it against the measured log current density (Figure 4).

Electrode characterization

The morphologies of the catalysts were examined using a Inspect F-50, Scanning Electron Microscope (SEM-FEI) at an accelerating voltage of 10 kV. Elemental composition was obtained by energy-dispersive X-ray microanalysis (EDS) coupled to SEM.

X-ray Photoelectron Spectroscopy (XPS) data were obtained with a SPECS GmbH system equipped with a hemispherical energy analyzer PHOIBOS 150 9MCD. A non-monochromatic Mg X-ray source was used with a power of 200 W and voltage of 12 kV. Pass energies of 50 and 20 eV were used for acquiring both survey and high-resolution spectra, respectively. These pass energies correspond to an Ag $3d_{5/2}$

FWHM of 1.6 eV and 1.0 eV. Peak position of the Ag $3d_{5/2}$ and Ag MNN peaks were used for instrument calibration. The Ag $3d_{5/2}$ and the Ag MNN peaks were found to be at 368.23 eV and at 895.74 eV binding energy positions, respectively.

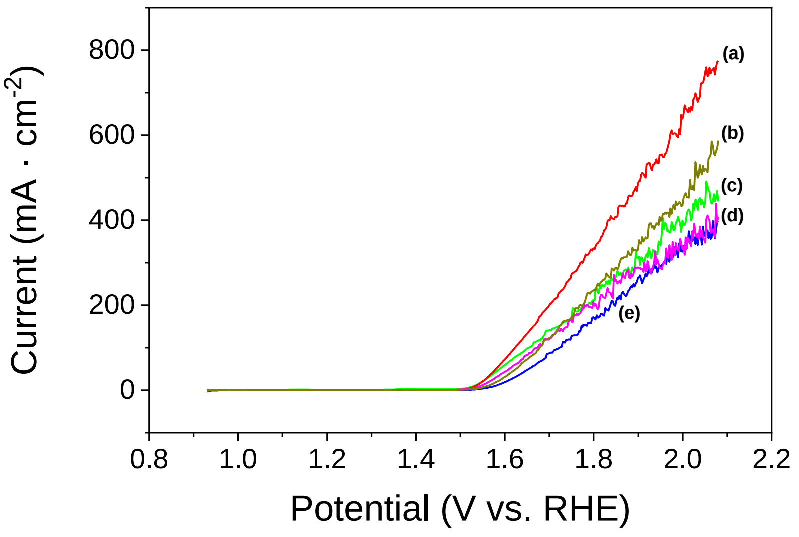

Figure S1. Linear sweep voltammograms in 1 M KOH solution of Co/Fc-modified electrodes with different Ferrocene loadings: (a) 18 µg, 0.25 mg·cm^{-2}; (b) 36 µg, 0.5 mg·cm^{-2}; (c) 9 µg, 0.125 mg·cm^{-2}; (d) 5.4 µg, 0.075 mg·cm^{-2}; (e) 3.6 µg, 0.05 mg·cm^{-2}. All graphite electrodes were prepared by drop casting of an ink containing $CoCl_2$ $6H_2O$ (36 µg, 0.5 mg·cm^{-2}) for the different catalyst configurations mixed with activated carbon Vulcan and Nafion and as described in procedures (Supporting information). Scan rate, 5 mV·s^{-1}.

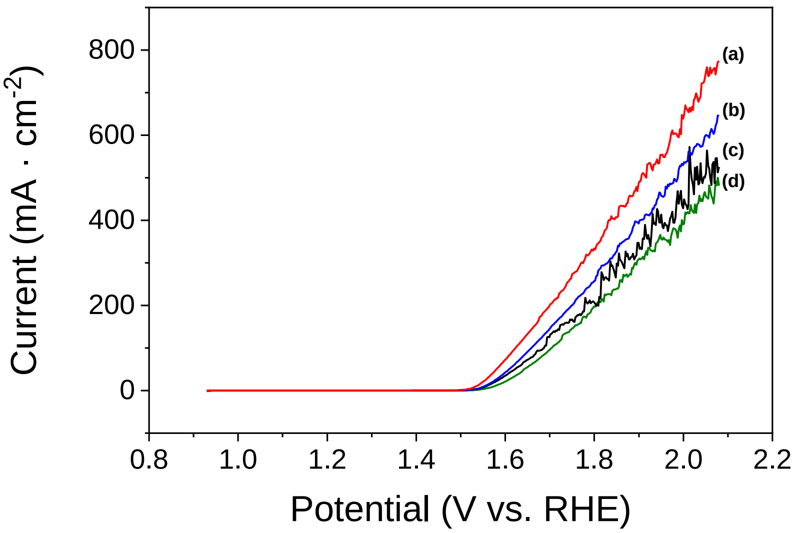

Figure S2. Linear sweep voltammograms in 1 M KOH solution of Co/Fc-modified electrodes with different $CoCl_2$ $6H_2O$ loadings: (a) 36 µg, 0.5 mg·cm^{-2}; (b) 72 µg, 1 mg·cm^{-2}; (c) 18 µg, 0.25 mg·cm^{-2}; (d) 9 µg, 0.125 mg·cm^{-2}. All graphite electrodes were prepared by drop casting of an ink containing ferrocene (18 µg, 0.25 mg·cm^{-2}) for the different catalyst configurations mixed with activated carbon Vulcan and Nafion and as described in procedures (Supporting information). Scan rate, 5 mV·s^{-1}.

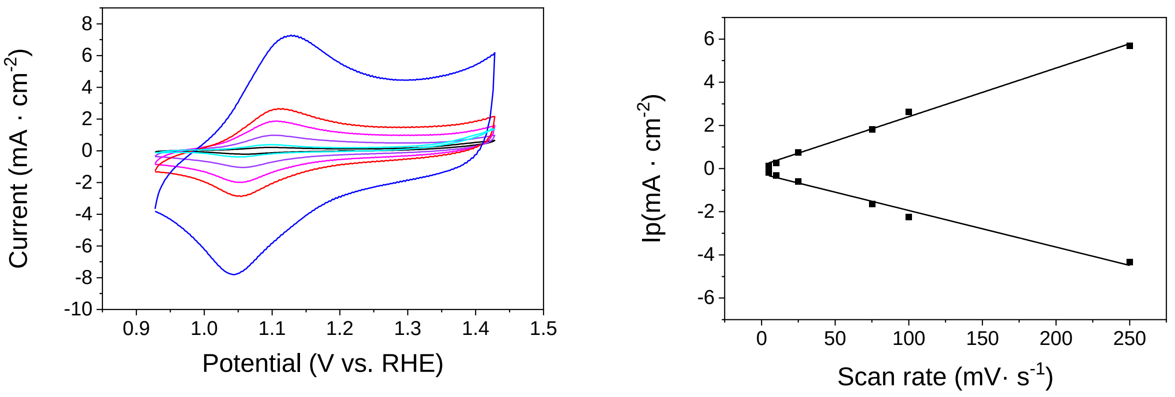

Figure S3. Cyclic voltammograms (CVs) in 1 M KOH solution of Co/Fc-modified electrode at scan rates of 250, 100, 75, 25, 10 and 5 mV·s^{-1}, respectively. Plot of peak current vs. scan rate.

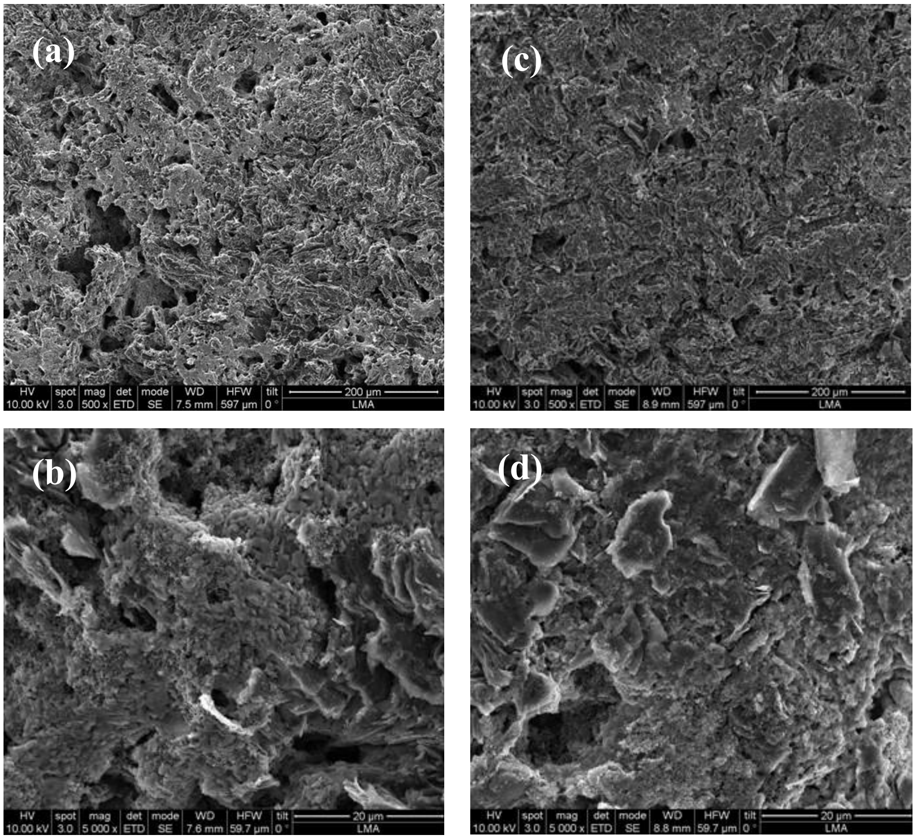

Figure S4. SEM images of Co/Fc-catalyst electrode surfaces at different magnifications: (a-b) as-prepared; (c-d) after OER performance in 1 M KOH solution.

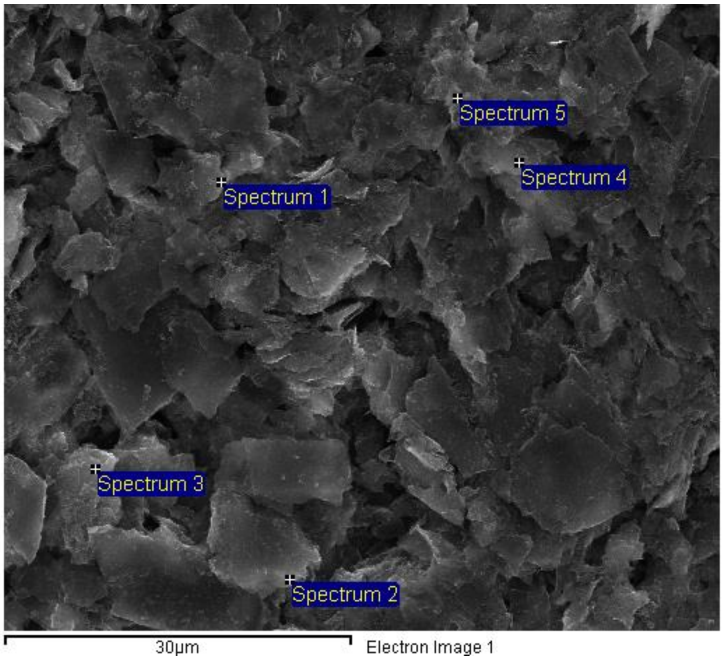

Spectrum	In stats.	C	O	F	Cl	K	Fe	Co	Total
Spectrum 1	Yes	67.06	15.07			13.14	1.05	3.68	100.00
Spectrum 2	Yes	69.53	16.43	2.87		7.88	0.46	2.83	100.00
Spectrum 3	Yes	82.16	12.66		0.41	3.06		1.71	100.00
Spectrum 4	Yes	87.05	7.63			1.78	0.56	2.98	100.00
Spectrum 5	Yes	69.64	12.72		1.09	10.92	0.87	4.78	100.00
Max.		87.05	16.43	2.87	1.09	13.14	1.05	4.78	
Min.		67.06	7.63	2.87	0.41	1.78	0.46	1.71	

All results in weight%

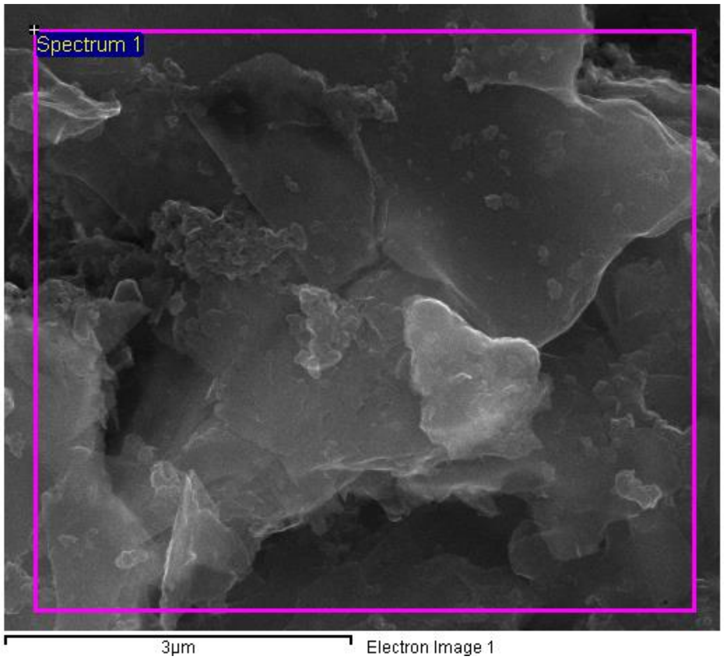

Spectrum	In stats.	C	O	Cl	K	Fe	Co	Total
Spectrum 1	Yes	72.85	14.31	0.26	8.90	0.92	2.76	100.00
Mean		72.85	14.31	0.26	8.90	0.92	2.76	100.00
Std. deviation		0.00	0.00	0.00	0.00	0.00	0.00	
Max.		72.85	14.31	0.26	8.90	0.92	2.76	
Min.		72.85	14.31	0.26	8.90	0.92	2.76	

All results in weight%

Figure S5. SEM images and EDX analysis of Co/Fc-catalyst electrode surfaces after OER performance in 1 M KOH solution.

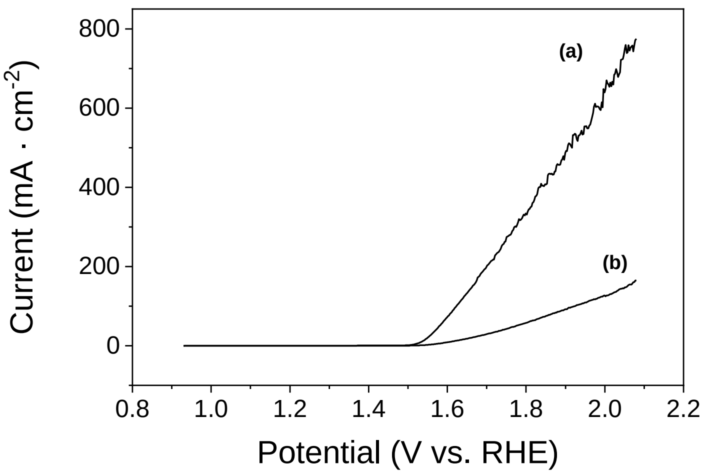

Figure S6. Linear sweep voltammograms in 1 M KOH solution of: (a) Co/Fc-modified electrode; (b) IrO_2 oxide-modified electrode. All graphite electrodes were prepared by drop casting of an ink containing the different catalyst configurations mixed with activated carbon Vulcan and nafion as described in procedure. Scan rate, 5 mV·s^{-1}

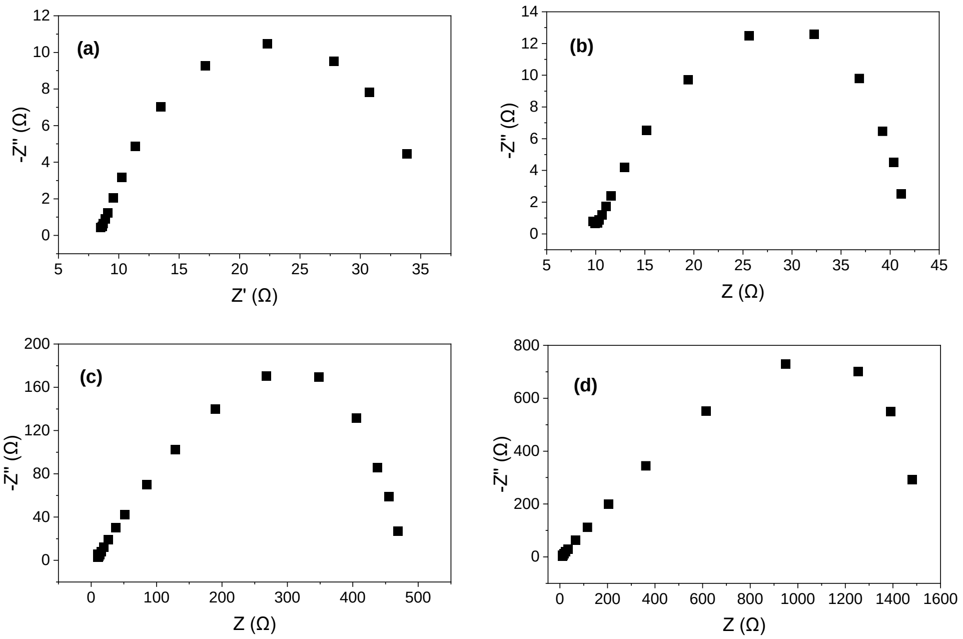

Figure S7. EIS measurements of a Co/Fc-catalyst (a); Co-catalyst (b); Fc-catalyst (c); Vulcan (d) electrodes in 1 M KOH.

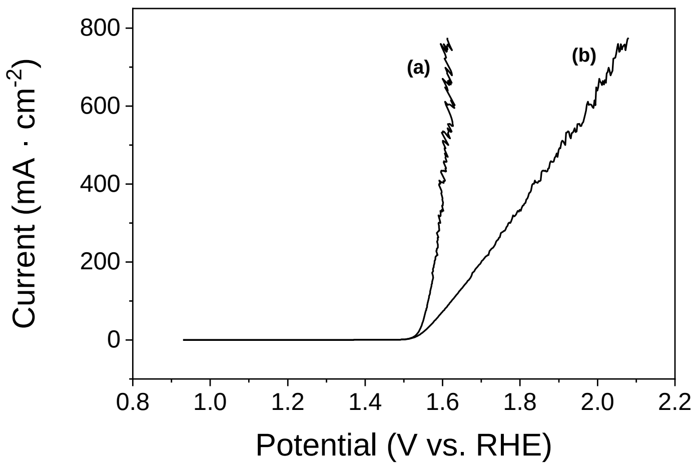

Figure S8. LSVs of a Co/Fc -catalyst electrode in 1 M KOH, recorded at a sweep rate of 5 $mV \cdot s^{-1}$ without (a) and with 100% iR drop compensations (b).

Co2p3/2 (eV)						
**Sample**	**position**	**FWHM**	**position**	**FWHM**	**Satellite**	**FWHM**
EL1	--	--	781.03	2.3	787.52	9
EL2	780.15	1.5	781.53	2.6	787.02	8.9
EL3	780.22	1.6	781.69	3	787.63	9

Table S1: Co 2p3/2 curve-fitted XPS binding energies for Fc/Co-modified electrodes: EL1, as-prepared without any electrochemical process; EL2, after CV as depicted in figure 1a; EL3, after further OER performance.

Fe2p3/2 (eV)						
**Sample**	**position**	**FWHM**	**position**	**FWHM**	**Satellite**	**FWHM**
EL1	709.64	2.7	712.65	2.3	718.85	3.6
EL2	709.98	3	712.12	3	718.24	5
EL3	709.74	2.5	713	3	717.8	5

Table S2: Fe2p3/2 curve-fitted XPS binding energies for Fc/Co-modified electrodes: EL1, as-prepared without any electrochemical process; EL2, after CV as depicted in figure 1a; EL3, after further OER performance.
